# Economic shocks, food insufficiency and mental health: Evidence from the COVID-19 pandemic

**DOI:** 10.1371/journal.pone.0344745

**Published:** 2026-03-12

**Authors:** Yuxuan Pan, Linlin Fan, Stephan Goetz

**Affiliations:** 1 Department of Agricultural Economics, University of Kentucky, Lexington, Kentucky, United States of America; 2 Department of Agricultural Economics, Sociology, and Education, The Pennsylvania State University, State College, Pennsylvania, United States of America; University College London, UNITED KINGDOM OF GREAT BRITAIN AND NORTHERN IRELAND

## Abstract

Millions of Americans experienced a sudden loss of income along with hunger early in the COVID-19 outbreak. Using Household Pulse Survey data from April 23, 2020 to March 29, 2021, we find the pandemic significantly impacted both food sufficiency and mental health, with food insufficiency having a larger negative impact on mental health than income loss. We do not find a statistically significant effect of unemployment on mental health. These findings were confirmed in various sensitivity analysis. We also discover heterogeneous effects of food insufficiency, unemployment, and income loss on mental health across different socioeconomic groups. Larger effects of food insufficiency were found in mortgage paying-households, among males, and in non-metro areas, and larger effects of income loss were found in rent paying-households, among females, and in non-metro areas. These results indicate the need for effective and timely policies targeting disadvantaged groups to maintain or improve their mental well-being, as well as food sufficiency, during future economic crises and public health emergencies.

## 1. Introduction

**Fig 1:**
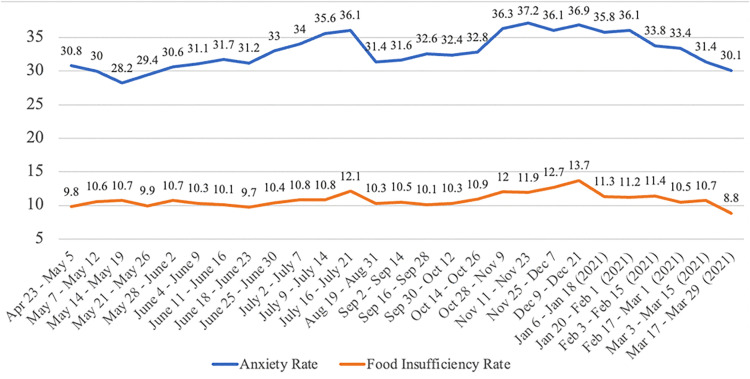
U.S. Average Food Insufficiency and Anxiety Rate (%) from HPS.

The COVID-19 pandemic and the associated economic collapse created unprecedented challenges for Americans. Many countries experienced disruptions in food acquisition and increased food insecurity during the pandemic [1–3], including the U.S. [4–6], where the share of food insecure households increased sharply. Before the pandemic, the overall U.S. food insecurity rate was 10.9%, the lowest it had been over the last twenty years [7]; during November and December 2020 (Fig 1) the food insufficiency rate rose to over 12% according to U.S. Census Data. Food insufficiency as defined by the U.S. Census Bureau is similar in concept to food insecurity as defined by the USDA-ERS, which indicates that a household is sometimes or often unable to acquire enough food over a predetermined period. At the same time, the COVID-19 pandemic greatly challenged households’ financial resources by upending labor markets, with many businesses shutting down amid massive job losses [8], particularly for individuals unable to work from home. The U.S. unemployment rate during the pandemic reached a level not seen since the Great Depression [9].

Mental health, another important concern during the downturns, has attracted policy makers’ interest because it not only imposes significant emotional and financial burdens on patients and their families, but also indirect costs to the public [10,11]. Fig 1 shows that over 30% of adults felt anxious in July 2020, and the anxiety rate was above 35% in November and December of 2020 [12]. This trend in poor mental health rates closely paralleled that of food insufficiency rates. Researchers have shown that food insecure individuals have worse mental health outcomes [13,14], while Wei et al. [15] found malnourished older adults are more depressed. Mental health and emotions are also negatively affected by job and income loss [16,17]. For example, unemployed people or people with income loss may worry about running out of financial resources in the future [18]. Compared to employed workers, working-aged unemployed individuals also had higher odds of suicide [19], and higher unemployment rates in turn led to higher suicide rates [20].

In this paper, we focus on anxiety (GAD-2) and depression (PHQ-2) as a specific indicator commonly used to assess mental health that is sensitive to general health and economic crises [21], and address three specific questions: how did unemployment, income loss and food insufficiency induced by Covid-19 affect mental health? Which predictor is the most important? In addition, which subgroups were hit hardest by the hardships induced by Covid-19 and experienced more mental disorders? We hypothesize that all three of these shocks—food insufficiency, income loss, and unemployment—increased the probability of experiencing mental disorders.

We employ linear probability models with instruments and use weekly household-level Household Pulse Survey (HPS) data from April 23, 2020 to March 29, 2021 to answer these questions. We have three main findings: First, food insufficiency has a larger negative impact on mental health than either income loss or unemployment. Surprisingly, we do not find a statistically significant effect of unemployment on mental health. On the one hand, unemployment may increase the likelihood of anxiety and depression as individuals worry about the future. On the other hand, unemployment may be associated with better mental health, possibly due to reduced concerns about contracting COVID-19 at work and the availability of generous unemployment insurance benefits during the pandemic [21]. Second, the negative impact of food insufficiency on mental health persists across all demographic groups. Subsample analyses show heterogeneous effects of food insufficiency and economic shocks across groups during the pandemic and reveal disadvantaged populations. Though previous literature shows that women are generally more vulnerable to have mental disorders [22,23], we find that males were more likely to be anxious if their households experienced food security problems. The larger negative effect of income loss on mental health were found in non-metro areas. These findings provide policy implications for identifying the most vulnerable groups in society and for providing prompt mental health assistance during shocks such as those created by economic or public health crises.

This paper contributes to the literature in three ways. First, previous researchers have started to study the effects of food insecurity [13,24–26] and labor markets shocks on mental health [16,17,27–29]. More recently, they have investigated the separate effects of food insecurity and employment on mental health within the context of COVID-19 [21,30,31]. However, there is limited research focusing on the joint causal effects of food sufficiency and economic shocks on mental health; we make a unique contribution by testing the hypotheses of a *simultaneous* relationship among food insufficiency, economic hardship and mental health during the COVID-19 pandemic. This allows us to provide policy implications by comparing the importance of each predictor in the relationship.

Second, earlier studies document associations between mental health, food insecurity, and economic shocks but do not establish their causal effects on mental health outcomes [21,25,29,31,32]. We contribute to the existing literature by addressing endogeneity between mental health and both food insufficiency and economic shocks, providing consistent estimates through an instrumental variable approach.

Lastly, prior research has primarily examined the average effects of food insecurity or economic shocks on mental health, using data from the U.S. Health and Retirement Study [23,29,32] or low-income-focused surveys [14,33,34]. With the richness of the nationally representative HPS data, we contribute to the existing literature by examining disproportionate impacts of food insufficiency and economic shocks on mental health among different socioeconomic groups. Our findings are especially relevant for policymakers in the context of food and health support programs.

## 2. Mechanisms

Our empirical analysis is guided by an economic conceptual framework grounded in household production theory [35,36]. We assume that households maximize utility, which depends positively on mental health, food consumption, and income. Within this framework, mental health is produced using inputs such as food sufficiency, income stability, and employment status. Below, we outline the key mechanisms linking these inputs to mental health.

First, food insufficiency reduces mental health production. Food insecurity generates both physiological strain and psychological stress, including anxiety driven by uncertainty and stigma associated with food acquisition [25,37].

Second, income loss and unemployment affect mental health through multiple channels. Reduced income and job loss impose financial strain, increasing stress and anxiety [18]. Unemployment may also lower self-esteem, further worsening mental health [31], and heightened uncertainty about future economic stability can amplify anxiety [8]. At the same time, unemployment increases available time in the household production function. Additional time may be allocated to activities that improve mental health, such as spending time with family and friends [38], and expanded unemployment insurance programs can mitigate financial stress, partially offsetting negative effects [21,31,39].

Finally, these mechanisms are heterogeneous. Stress exposure and coping strategies differ by gender, leading to variation in mental health outcomes [22]. Geographic disparities, such as differences in access to mental health services between metropolitan and non-metropolitan areas, also contribute to unequal mental health outcomes [40].

## 3. Data and methods

### 3.1. Data source

The Census Bureau initiated the HPS on April 23, 2020, as a real-time survey of American households, through an online platform to measure social and economic impacts of the COVID-19 pandemic. The HPS provides insights into how employment, health, food security, housing, childcare, and education changed during the COVID-19 pandemic based on a nationally representative revolving sample. Our analysis uses 27 weeks of household-level public-use data files, over the survey periods April 23, 2020 to March 29, 2021. Results before July 22, 2020 of the survey were released weekly. All later results were released every two weeks and, in order to maintain continuity, the HPS continues to call these two-week collection periods “weeks” [12]. As the HPS replaces the survey sample in every interviewing week, we cannot use panel regression methods and instead use a pooled cross-sectional dataset in the regression. We apply the HPS household weights to account for nonresponse and coverage of the demographic characteristics of the interviewed persons in the household to obtain household-level estimates [41]. The HPS household weights produce a distribution of households by key demographic factors that align well with American Community Survey (ACS) household estimates.

### 3.2. Variables

#### 3.2.1. *Mental health.*

The Household Pulse Survey (HPS) assesses the frequency of anxiety and depression symptoms. For anxiety, we use the validated two-item Generalized Anxiety Disorder scale (GAD-2) [42]. According to HPS definitions, a respondent is considered to have anxiety symptoms if they report feeling nervous, anxious, or unable to control worrying “more than half the days” or “nearly every day” during the past week. For depression, we use the validated two-item Patient Health Questionnaire (PHQ-2) [43]. Respondents are considered to have depression symptoms if they report having little interest or pleasure in doing things, or feeling down, depressed, or hopeless “more than half the days” or “nearly every day” during the past week. Consistent with prior research, we consider a score of 3 or higher on the PHQ-2 to indicate major depressive disorder [43], and a score of 3 or higher on the GAD-2 to indicate generalized anxiety disorder [42].

#### 3.2.2. *Food insufficiency and economic shocks.*

Food insufficiency is our first independent variable of interest. Based on HPS definitions, members in households are food insufficient if they “sometimes” or “often” do not have enough to eat. Food insufficiency could affect mental health through several possible mechanisms. First, consumption of cheap food and inadequate intake of critical nutrients contributes to stress and anxiety [25]. Second, inadequate access to food leads to anxiety from uncertainty about future food availability [30,44]. Third, food insufficiency leads to worse physical health, such as diabetes and obesity [24], which influences one’s mental health.

Mental health also depends on income and employment. Income loss may increase stressful life events [19] and unstructured time [29]; a binary answer in the HPS indicates whether anyone in the household has experienced a loss of employment income since March 13, 2020. In addition, unemployment has a detrimental effect on mental health by decreasing financial resources, lowering self-esteem and reducing sense of fulfillment and purpose [10]; this variable shows the respondent’s current employment status in the last 7 days (1 = unemployed, 0 = employed). It is important to note that food insufficiency reflects a contemporaneous household condition at the time of the survey, rather than a change relative to a pre-pandemic baseline. In contrast, income loss is explicitly defined as a change since March 13, 2020, and unemployment captures current employment status relative to prior employment. Therefore, these measures differ conceptually in terms of timing and persistence, which should be considered when interpreting and comparing estimated magnitudes.

In addition, to mitigate concerns on the endogeneity of unemployment, we only focus on the effect of involuntary unemployment, i.e., the sample that only includes respondents who currently do not have a job because of exogenous reasons: *“8) My employer experienced a reduction in business (including furlough) due to the coronavirus pandemic; 10) My employment closed temporarily due to the coronavirus pandemic; 11) My employment went out of business due to the coronavirus pandemic;”.* Otherwise, if the respondents are unemployed because they are fearful of working or other mental health-related reasons, a biased coefficient may arise in the estimation. For example, the survey asks, “*Main reason for not working for pay or profit*”, if the respondent chooses the voluntary unemployment answer which may be highly correlated with mental health, such as “*I did not want to be employed at this time*” or “*I am/was caring for someone with coronavirus symptoms*”, the unemployment variable would be more endogenous in the estimation. Hence, we delete such sample.

#### 3.2.3. *Covariates.*

The HPS also provides control variables to capture respondent and household characteristics. They are respondents’ age, gender, race, marital status, Hispanic origin, education, household size, number of children and total household annual income level in 2019. These variables are commonly used in other research to analyze the impact of food sufficiency and employment on mental health (e.g., [14,45]). For example, we add age and age square to control for the U-shaped relationship between age and life satisfaction [46]. Gender is included because of the difference between men and women in mental well-being from previous literature (e.g., [10,47]). Controlling for income is also important because it is a crucial determinant of health [24].

#### 3.2.4. *State-level variables.*

The timing of responses to the arrival of the coronavirus varied across states, and state-level time-variant policies are likely to be correlated with household mental health due to consumption and economic shocks. Therefore, we control for two sets of confounding state-level variables in our regression. First, inspired by Restrepo et al. [41], we include Google Mobility Trends at the state level, which show the daily change in visits to places such as residential (Google LLC.) relative to the baseline pre-pandemic period (January 3 to February 6, 2020). Similar to Restrepo et al. [41], we use the state-level visits for the first day in each HPS week (e.g., the visit value for April 23 is used for the HPS week 1 April 23 to May 5). Second, we include indicator variables from HealthData.gov to indicate whether the state has stay-at-home and non-essential-business closure orders in a given week. Stay-at-home orders and restrictions on entertainment activities increase levels of stress, anxiety and depression in the population [48].

### 3.3. Empirical analysis

This paper formally estimates the effects of shocks (food insufficiency, income loss, and unemployment) on mental health and compares their magnitudes during the pandemic. Identifying the causal effect is not straightforward due to the well-established issues of reverse causality between mental health and shocks (food insufficiency, income loss, and unemployment). For example, people with more mental health disorders are more likely to earn less, be food insufficient and unemployed. In addition, other unobserved variables influence both economic shocks, food insufficiency and mental health. Therefore, a simple linear regression model (OLS) will provide inconsistent estimates for the parameters of interest. To correct for endogeneity introduced above, we employ a linear probability model with instrumental variables (referred to as LPM-IV). In the first step, we estimate a LPM model for each endogenous indicator variable as functions of instruments and other control variables. Then we regress the mental health indicators (*PHQ-2 and GAD-2*) on the predicted values of endogenous variables and the covariates. The LPM-IV approach employed in this study is illustrated by the first and second stage as follows:


$$\begin{array}{c} \;{Shocks}_{ist}={𝐙}_{ist}^{\prime }\pi_{\text{1}}+{𝐗}_{ist}^{\prime }\pi_{2}+{𝐒}_{st}^{\prime }\pi_{3}+t+\lambda_{s}+\lambda_{s}𝐭+{𝐯}_{ist} \end{array}$$ (1)

$$\begin{array}{c} {\;Mental\;Health}_{ist}={\hat{Shocks}}_{ist}\beta_{\text{1}}+{𝐗}_{ist}^{\prime }\beta_{2}+{𝐒}_{st}^{\prime }\beta_{3}+{t+\lambda_{s}+\lambda_{s}𝐭+\in }_{ist} \end{array}$$ (2)

where ${Mental\;Health}_{ist}$ is a binary variable that indicates whether the respondent in household *i* experienced a mental health disorder (anxiety/depression) in state *s* and week *t*, ${Shock}_{ist}$ is a set of indicators measuring whether *t*he members in household *i* experience food insufficiency, whether anyone in a household had a loss in income or the representative household member (respondent) is currently unemployed. $X_{ist}$ and $S_{st}$ include household level and time-varying state-level characteristics, as described in section 3.2 and summarized in Table 1. ***Z***_***is*t**_ are the IVs which are discussed in detail in the next section. Time trend *t* is included to capture the trajectory of mental health over time. State fixed effect $\lambda_{s}$ is included to control for time-invariant state-level characteristics. As Restrepo et al. [41] argued, policy responses to the spread of COVID-19 that related to food and mental health hardship and economic shocks have also varied within states over time. To control for this confounding factor, we also include state-specific trends $\lambda_{s}𝐭$. $\in_{ist}$ and ${𝐯}_{ist}$ are error terms. Standard errors are clustered at the state level in order to control for arbitrary correlation among observations from the same state.

**Table 1:** Summary statistics for variables used in the regression.

	All		Mental Disorder = yes		Mental Disorder = no		
	mean	sd	mean	sd	mean	sd	p-value
**Dependent Variable**							
Anxiety (GAD-2)	0.339	0.473	0.879	0.327	0		
Depression (PHQ-2)	0.263	0.440	0.680	0.466	0		
**Shocks**							
Food Insufficiency	0.097	0.296	0.174	0.379	0.049	0.216	<0.001
Income loss	0.479	0.500	0.587	0.492	0.411	0.492	<0.001
Unemployment	0.108	0.311	0.149	0.356	0.083	0.275	<0.001
**Household Characteristics**							
Household size	3.010	1.543	2.994	1.560	3.021	1.533	<0.001
Number of Children	0.804	1.118	0.782	1.110	0.819	1.123	<0.001
Income < $25,000	0.105	0.307	0.145	0.352	0.080	0.271	<0.001
Income $25,000-$49,999	0.211	0.408	0.251	0.434	0.186	0.389	<0.001
Income $50,000 - $99,999	0.300	0.458	0.298	0.457	0.301	0.459	0.211
Income $100,000-$199,999	0.233	0.423	0.184	0.388	0.264	0.441	<0.001
Income > $200,000	0.080	0.271	0.051	0.221	0.098	0.297	<0.001
**Respondent Characteristics**							
Age	42.267	12.384	40.106	12.249	43.627	12.274	<0.001
Age square	1939.86	1072.30	1758.48	1035.00	2054.01	1079.61	<0.001
Male	0.512	0.500	0.454	0.498	0.549	0.498	<0.001
Married	0.522	0.500	0.442	0.497	0.572	0.495	<0.001
Hispanic	0.154	0.361	0.164	0.370	0.148	0.355	<0.001
White	0.763	0.425	0.760	0.427	0.765	0.424	0.016
Black	0.124	0.329	0.127	0.333	0.122	0.327	<0.001
Asian	0.056	0.230	0.046	0.209	0.063	0.243	<0.001
Other	0.057	0.231	0.067	0.249	0.051	0.219	<0.001
Less than high school	0.016	0.127	0.018	0.132	0.016	0.124	0.018
High school graduate or equivalent	0.291	0.454	0.302	0.459	0.284	0.451	<0.001
Some college or Associates degree	0.303	0.460	0.332	0.471	0.285	0.451	<0.001
Bachelor’s or graduate degree	0.389	0.488	0.348	0.476	0.415	0.493	<0.001
**State Characteristics**							
Residential mobility	11.653	3.777	11.625	3.723	11.671	3.810	0.009
Stay-at-home order	0.149	0.356	0.148	0.355	0.149	0.357	0.506
Non-essential-business order	0.178	0.382	0.171	0.377	0.182	0.386	<0.001
Observations	1,039,923		383,130		656,793		

*Notes:* The group reporting mental disorders includes respondents who scored 3 *or* higher on the GAD-2 scale (indicating anxiety symptoms more than half the days or nearly every day) or scored 3 or higher on the PHQ-2 scale (indicating depression symptoms more than half the days or nearly every day), or both. These cutoffs represent validated thresholds for generalized anxiety disorder and major depressive disorder, respectively. Sample weights are used to adjust the summary statistics. The t-test column reports the p-values from tests comparing households with and without mental disorders, indicating whether the differences between the two groups are statistically significant.

#### 3.3.1. *Lewbel instrumental variables.*

A valid instrument must fulfill two important conditions: (i) relevance, i.e., it has to be significantly correlated with the endogenous variable and, (ii) the exclusion restriction, i.e., the instrument has to affect mental health only through food insufficiency, income loss, or unemployment [49–51]. With valid instruments, the endogeneity issue could be addressed, but such traditional instruments are difficult to find to satisfy both conditions. Subramanian and Deaton [52] have shown that it is extremely difficult to find instruments that are correlated with *food* but uncorrelated with the error (*mental health*). Weak instruments may actually produce more biased estimates than ordinary least squares [53]. Similar identification challenges arise in unemployment-health literature. Though plant closures and mass workforce reductions provide commonly used exogenous variation in unemployment [54,55], these events occur infrequently and disproportionately affect particular populations, such as blue-collar workers, thereby constraining generalizability [56].

To obtain instruments that avoid the pitfalls mentioned above, we employ the Lewbel [57] IV approach, which allows us to identify the effects of interest by accounting for unobservable heterogeneity without relying on external instruments, particularly when external instruments are unavailable, difficult to find, or insufficient. The Lewbel IV estimator has become increasingly common in recent years (e.g., [58,59]). The Lewbel IV approach exploits heteroskedasticity in the first-stage error term to construct internal instruments from observed exogenous variables, allowing identification even in the absence of traditional external instruments. A key advantage is that it can transform a just-identified model into an overidentified one, enabling specification tests and potentially improving efficiency. Compared with standard IV methods, Lewbel IV often yields more precise estimates with smaller standard errors, while still allowing consistent identification of structural parameters in models with endogenous regressors.

#### 3.3.2. *Assumptions.*

The validity of the Lewbel IV approach relies on two key assumptions. Let Z  denote a set of exogenous variables included in the first-stage regression. Lewbel (2012) shows that $(\text{Z}-\bar{\text{Z}}hat\text{v}$ can serve as valid instruments under the following conditions:


$$𝐀1:\;\text{Cov}\;\left(\text{Z},{\text{v}}^{\text{2}}\right)\neq \text{0}$$

𝐀2: Cov (Z,vϵ)=0

Assumption A1 requires that the exogenous variables Z (e.g., gender, household size, education) be correlated with the variance of the first-stage error term. In other words, the unobserved shocks affecting food insufficiency and economic disruptions must exhibit heteroskedasticity across household types defined by Z. This condition is plausible in the COVID-19 context, as pandemic impacts varied systematically across demographic groups. For example, household structure influenced food access constraints (multi-generational vs. single-person households managing bulk purchases and multiple dependents differently), and gender shaped labor market vulnerability (female-dominated service sectors experienced higher job losses than male-dominated construction), generating differential exposure to unobserved shocks and thus heteroskedasticity.

Assumption A2 requires that the exogenous variables Z be uncorrelated with the product of the first- and second-stage error terms. This implies that demographic characteristics must not share unobserved determinants with both the endogenous regressors (food insufficiency and economic shocks) and mental health outcomes, conditional on controls. Following prior studies (e.g., [60,61]), we select socio-demographic variables (household size, gender, education, age, marital status) as exogenous instruments and include extensive controls for household income, demographic characteristics, state-level pandemic policies, mobility trends, state and time fixed effects, and state-specific time trends. These controls mitigate concerns that Z  is correlated with unobserved confounders. We provide empirical tests supporting the validity of the Lewbel IV strategy in the Results section.

## 4. Results

### 4.1. Descriptive statistics

Table 1 shows summary statistics for the entire sample, along with separate tabulations for those reporting and not reporting mental health disorders. Most variables from the HPS are categorical. Table B in S1 File presents descriptions of all variables. For the dependent variable, 34% of the entire sample feel anxious more than half the days or nearly every day and 26% of the entire sample feel depressed more than half the days or nearly every day. 10% of respondents report household food insufficiency in the overall sample. Compared to the no-mental disorder group, the group with mental disorder has a higher share of hunger with 17% reporting food insufficiency. In the overall sample, 48% of respondents report someone in their household has experienced a loss in income, and 11% of the sample remain unemployed currently. Compared to the group with no mental disorder symptoms, the group with mental disorder has a larger share of females (55% vs 45%), Hispanics (16% vs 15%), and not-married populations (56% vs 43%), and a smaller share of Asians (5% vs 6%) and highly-educated populations with a bachelor’s or graduate degree (35% vs 42%). On average, due to the stay-at-home order of many states, visits to residential places were higher than the pre-pandemic baseline (January 3 to February 6, 2020).

### 4.2. Causal effects of food insufficiency and economic shocks on mental health

Table 2 presents OLS regression results, and results of the LPM-IV. The magnitudes of the OLS coefficients on food insufficiency and income loss are similar to those from the LPM-IV, but the coefficient on unemployment becomes insignificant once endogeneity is addressed. First of all, the effect of food insufficiency is positive, which means that if members of a household sometimes or often do not have enough to eat, then the respondent has a higher probability of reporting mental disorders. This correlation is as expected: being food insufficient is associated with poor mental health. People are also more likely to report anxiety or depression if anyone in the household has experienced a loss of income. However, we did not find statistically significant evidence of an effect of unemployment on mental health.

**Table 2:** Estimates of OLS and LPM-IV models of mental disorders.

	OLS		LPM-IV	
	(1)	(2)	(3)	(4)
Dep. Variable:	Anxiety (GAD-2)	Depression (PHQ-2)	Anxiety (GAD-2)	Depression (PHQ-2)
Food Insufficiency	0.255***	0.252***	0.267***	0.236***
	(0.005)	(0.006)	(0.012)	(0.012)
Income Loss	0.112***	0.086***	0.128***	0.109***
	(0.003)	(0.004)	(0.008)	(0.009)
Unemployment	0.051***	0.064***	−0.017	−0.008
	(0.004)	(0.004)	(0.024)	(0.018)
Household size	−0.0004	0.0003	−0.001	−0.001
	(0.001)	(0.001)	(0.001)	(0.001)
Number of kids	−0.011***	−0.016***	−0.010***	−0.015***
	(0.001)	(0.001)	(0.001)	(0.001)
Income 25,000–49,999	0.010**	0.012***	0.007*	0.008*
	(0.004)	(0.004)	(0.004)	(0.004)
Income 50,000–99,999	−0.008***	−0.018***	−0.011***	−0.024***
	(0.003)	(0.003)	(0.004)	(0.004)
Income 100,000–199,999	−0.042***	−0.049***	−0.045***	−0.055***
	(0.004)	(0.003)	(0.004)	(0.004)
Income >200,000	−0.067***	−0.071***	−0.069***	−0.076***
	(0.006)	(0.003)	(0.007)	(0.006)
Age	0.0004	−0.004***	0.0003	−0.004***
	(0.0004)	(0.001)	(0.0004)	(0.001)
Age square	−0.00005***	0.00001	−0.00005***	0.00001
	(0.00001)	(0.00001)	(0.00001)	(0.00001)
Male	−0.083***	−0.030***	−0.082***	−0.029***
	(0.002)	(0.002)	(0.002)	(0.002)
Married	−0.034***	−0.052***	−0.035***	−0.053***
	(0.002)	(0.002)	(0.002)	(0.002)
Hispanic	−0.038***	−0.034***	−0.038***	−0.033***
	(0.004)	(0.004)	(0.004)	(0.004)
Black	−0.059***	−0.038***	−0.058***	−0.034***
	(0.004)	(0.005)	(0.004)	(0.006)
Asian	−0.079***	−0.030***	−0.077***	−0.028***
	(0.005)	(0.004)	(0.005)	(0.005)
Other Race	0.013***	0.015***	0.013***	0.016***
	(0.004)	(0.004)	(0.004)	(0.004)
HS degree or GED	−0.002	0.001	−0.003	−0.001
	(0.010)	(0.009)	(0.010)	(0.009)
Some college/AA degree	0.031***	0.024**	0.030***	0.020**
	(0.012)	(0.010)	(0.012)	(0.010)
Bachelors’/Graduate degree	0.029**	−0.009	0.028**	−0.015
	(0.011)	(0.010)	(0.011)	(0.010)
Residential Mobility	−0.001	0.002***	−0.0004	0.002***
	(0.001)	(0.0005)	(0.0005)	(0.0005)
Stay-at-home order	−0.005	−0.001	−0.005	−0.0003
	(0.010)	(0.006)	(0.009)	(0.006)
Non-essential-business	−0.005	−0.002	−0.005	−0.002
	(0.007)	(0.005)	(0.007)	(0.004)
Time trend	0.002***	0.003***	0.001***	0.002***
	(0.00008)	(0.00007)	(0.0002)	(0.0001)
Constant	0.380***	0.379***	0.387***	0.389***
	(0.014)	(0.016)	(0.014)	(0.016)
*Statistics:*				
First Stage F: Food Insufficiency			6198 (<0.001)	
(P-Value)				
First Stage F: Income Loss			14075	
(P-Value)			(<0.001)	
First Stage F: Unemployment			792	
(P-Value)			(<0.001)	
Breusch-Pagan: Food Insufficiency			30298	
(P-Value)			(<0.001)	
Breusch-Pagan: Income Loss			980	
(P-Value)			(<0.001)	
Breusch-Pagan: Unemployment			13532	
(P-Value)			(<0.001)	
Hansen-J Statistics (P-Value)			38 (0.24)	36 (0.34)
Observations	1,039,923			

*Notes:* All estimation results are adjusted by sampling weights. The dependent variable for *anxiety* is a binary indicator equal to 1 if the respondent scored 3 or higher on the GAD-2 scale, indicating anxiety symptoms for more than half the days or nearly every day. The *depression* variable is defined similarly, utilizing a threshold of 3 or higher on the PHQ-2 scale. Breusch-Pagan χ² tests reject homoskedasticity in all first-stage regressions (p < 0.01), supporting assumption A1. Hansen J overidentification test does not reject valid instruments, supporting assumption A2. First-stage F-statistics test instrument strength and relevance. Standard errors are clustered at state level. ***, **, and *, denote significance at the 1%, 5%, and 10% levels, respectively.

Additionally, the effect of food insufficiency is larger than the effect of income loss (column 3 and 4). To be specific, if a household does not have enough to eat, the probability of anxiety (depression) is 27 (24) percentage points higher. If someone in a household has experienced income loss, the respondent has a 13 (11) percentage points higher probability of reporting anxiety (depression). An important caveat to this magnitude comparison is that our food insufficiency measure may capture both households experiencing new food insufficiency during the pandemic and those with chronic, pre-existing food insufficiency, while the income loss and involuntary unemployment explicitly measure changes in status. To examine this heterogeneity, we conducted a supplementary analysis using an HPS question about pre-pandemic food insufficiency status; a binary indicator equal to 1 if the household reported “sometimes” or “often” not having enough to eat prior to March 13, 2020, and 0 otherwise. Results in Table A3 in S1 File show that persistent food insufficiency (food insufficient both before and during the pandemic) has substantially larger effects than new food insufficiency (food insufficient during but not before the pandemic): 49.1 versus 29.1 percentage points for anxiety and 42.7 versus 23.8 percentage points for depression. This pattern is consistent with research showing persistent food insecurity has more severe consequences than transitory food insecurity [62].

The results also show that low-income individuals have a higher probability of reporting mental disorders than high-income individuals. However, highly educated respondents are more likely to report anxiety than those without a high school degree. Hispanics and Blacks are less likely to report anxiety and depression compared to Whites. At the state level, increased residential mobility is associated with a rise in depression. Mandated reductions in visits to outdoor spaces may make it more difficult for people to relieve stress through social activities [63,64].

Table 2 also presents statistical evidence supporting the validity of the Lewbel IV approach. Breusch-Pagan χ² tests reject homoskedasticity in all first-stage regressions (p < 0.01), supporting assumption A1. Hansen J overidentification test do not reject valid instruments, supporting assumption A2. In addition, the first-stage F-statistics for all three endogenous variables exceed 10, indicating that the instruments are strong. Hence, we find no direct evidence against the validity of the instruments.

### 4.3. Subgroup analysis

To identify vulnerable populations that are more affected by economic shocks and food insufficiency, we evaluate the effects in different subgroups in Table 3. In general, the impact of food insufficiency is larger than that of economic shocks for all groups and significant statistically, while the coefficient of involuntary unemployment becomes negative in some subgroups.

**Table 3:** Heterogenous effects of LPM-IV.

	Mortgage	Rent	Male	Female	Non-Metro	Metro
*Dep. Var: Anxiety (GAD-2)*						
Food Insufficiency	0.279***	0.240***	0.265***	0.261***	0.268***	0.263***
	(0.014)	(0.018)	(0.014)	(0.019)	(0.018)	(0.016)
Income loss	0.108***	0.129***	0.112***	0.143***	0.150***	0.099***
	(0.011)	(0.013)	(0.013)	(0.010)	(0.013)	(0.017)
Unemployment	0.006	−0.029	0.019	−0.116**	−0.020	−0.016
	(0.020)	(0.021)	(0.025)	(0.046)	(0.024)	(0.018)
*Dep. Var: Depression (PHQ-2)*						
Food Insufficiency	0.260***	0.201***	0.234***	0.234***	0.247***	0.220***
	(0.021)	(0.015)	(0.015)	(0.015)	(0.015)	(0.014)
Income loss	0.108***	0.099***	0.091***	0.141***	0.135***	0.088***
	(0.009)	(0.012)	(0.015)	(0.009)	(0.012)	(0.023)
Unemployment	0.002	0.002	0.030	−0.079**	−0.010	−0.006
	(0.018)	(0.019)	(0.025)	(0.031)	(0.019)	(0.017)
Observations	624,097	325,793	479,698	644,514	720,207	404,005

*Notes:* All estimation results are adjusted by sampling weights. The dependent variable for *anxiety* is a binary indicator equal to 1 if the respondent scored 3 or higher on the GAD-2 scale, indicating anxiety symptoms for more than half the days or nearly every day. The *depression* variable is defined similarly, utilizing a threshold of 3 or higher on the PHQ-2 scale. Standard errors are clustered at state level. ***, **, and *, denote significance at the 1%, 5%, and 10% levels, respectively.

We first examine the mortgage and rent payment subsamples. We select these subgroups because home ownership has been found to be an important determinant for employment, food sufficiency, and mental health. For instance, research shows that people who do not own their home tend to be employed in jobs with lower possibilities of telecommuting [65], hence, they are more likely to be exposed to the virus and be unemployed due to lock-down. We expect the effect of economic shocks on mental health is larger for households without home ownership. HPS asks whether the household owns a home with mortgage or rents it. As many households did not answer this question in the survey, we have fewer observations in these two groups. Both groups are significantly affected by food insufficiency and economic shocks. Compared to food sufficient households, the probability of the food insufficient mortgage paying group being anxious increases by 28 percentage points. In contrast, this number is only 24 for the rent- paying group. When examining economic shocks, income loss also causes rent paying respondent households to report anxiety with higher probability than mortgage-paying counterparts. Examining the assistance program related statistics of these two groups (Table A1 in S1 File), we find that rent payers have a higher share receiving Supplemental Nutrition Assistance Program (SNAP) than those with mortgages. It could be that although the rent paying group experiences food insufficiency, they are eligible for food support programs and receive free food, and so the hunger shock is not as large as that experienced by mortgage payment groups [66].

The second comparison verifies if there is a difference between male respondents and female respondents. Previous research indicates gender gaps in household food security problems and mental health disorders [22,23,47]; women were affected by the COVID more than men [67]. Our subgroup comparison shows the effects of food insufficiency on anxiety were stronger for men during the pandemic. Columns 3 and 4 indicate that mental disorders increase with food insufficiency and job loss among male workers, with the probability of experiencing anxiety increasing by roughly 27 percentage points if the respondent’s family experienced a food hardship, and 11 percentage points if it had an income loss. The difference between gender can also be seen in Table A1 in S1 File: females receive more mental health care and SNAP, which will reduce the effect of hunger on anxiety. However, the effect of involuntary unemployment on mental health disorder is negative for females. This means that although a female respondent lost her job, involuntary unemployment is associated with a 12-percentage point decrease in the likelihood of being anxious and an 8 percentage point decrease in the likelihood of being depressed.

Third, previous research shows households in metropolitan areas are with higher exposure to the pandemic and experience bigger reductions in economic activities than non-metro areas [1]. For this purpose, we compare different effects of food insufficiency and economic shocks between households living in the top 15 metropolitan areas (metro areas) and households not living in those areas (non-metro areas). Results in Table 3 show the effect of food insufficiency is significant on both groups but there is a bigger effect for the non-metro households. It is possible that healthcare access prevents residents in non-metro areas from receiving adequate mental care. For example, we find in our data that the share receiving mental health care in the non-metro group is smaller than in the metro group although more residents in the non-metro area receive free food (an indicator in HPS to show whether anyone in a household received a free meal or free groceries during the last 7 days). to reduce hunger (Table A1 in S1 File).

### 4.4. Robustness checks

We include robustness checks in Table A2 in S1 File. Columns 1 and 2 present results from regressions where food insufficiency and economic shocks are included separately. In column 3, we exclude household income and residential mobility in the specification. Column 4 shows the results when we add COVID cases and vaccine rate as state-level controls. Note that we do not observe the residence of the respondents other than the states they live in, so we are unable to match COVID vaccination and cases at the county level. Last, since the interaction of decisions and intra-household allocations regarding mental health, food, and employment within a household was important during the pandemic, especially for households with multi-decisionmaker [60,68]. We conduct a robustness check by separately analyzing households with a single individual and those with more than one individual in the last two columns. The coefficient estimates for the variables of interest remain consistent across all robustness checks. These results further support the validity of the Lewbel instruments, as household size is excluded from the instrument set in Columns 5 and 6 of Table A2 in S1 File and the estimates remain stable.

## 5. Discussion and conclusion

Adults in the United States faced severe food and mental health hardships throughout the pandemic. This study analyzes the effect of food insufficiency and economic shocks on mental health disorders during the pandemic and compares the magnitudes of these shocks on mental health. Based on household-level survey data, the results show that food insufficiency and income loss significantly impact mental health. In addition, we find a larger impact from food insufficiency than income loss on mental health. Food insufficient households were more likely to experience anxiety (depression) symptom by 27 (24) percentage points. The results suggest that individuals who experienced income loss were 13 (11) percentage points more likely to experience anxiety (depression). However, we do not find a statistically significant effect of involuntary unemployment on mental health. Based on subgroup regressions, we find males, mortgage payers and non-metro area populations had higher probabilities of reporting anxiety disorder when they were food insufficient, compared to their counterparts.

Our findings call for effective and timely policies to guard against the effects of future public health emergencies and economic crises like the COVID-19 pandemic and similar situations. First, we find food insufficiency has a larger impact on mental health than income loss. However, as noted in our results, this comparison should be interpreted recognizing that food insufficiency during the pandemic included both newly food insufficient households and chronically food insufficient households. Supplementary analysis (Table A3 in S1 File) reveals persistent food insufficiency has substantially larger effects (49 percentage points for anxiety; 43 percentage points for depression) than new food insufficiency (29 percentage points for anxiety; 24 percentage points for depression). Distinguishing chronic from transient food insufficiency is essential for policy design, as chronically food insufficient households may require longer-term, sustained support through programs, while newly food insufficient households may benefit from short-term emergency assistance to prevent their food insecurity from becoming chronic [62]. The government made significant policy changes in response to COVID-19, such as a 15% increase in benefits for those eligible for SNAP and the distribution of Pandemic Electronic Benefits for grocery shopping [69], however, our results in Table A4 in S1 File show that receiving food assistance does not eliminate the effect of hunger on anxiety or depression. For example, receiving free food or SNAP only reduced the probability of anxiety by 5 percentage points if the household was food insufficient. Although these results cannot be interpreted as causal effects due to the endogeneity of receiving benefits, they still call for immediate targeting strategies to identify the most impacted populations with mental health problems due to hunger. This calls for support from the federal government to improve the efficiency and effectiveness of emergency food and health service provision to incorporate future disaster, such as enhancing collaboration across sectors [70].

In addition, many social benefit programs currently target low-income, low-educated, and other disadvantaged groups [71–73], but our subgroup analysis shows that mortgage-paying families had a higher probability of reporting anxiety or depression disorder when they were food insufficient compared to the rent-paying group. Due to asset tests restrictions, some mortgage-paying families are not able to receive food assistance programs to reduce hunger and stress [66]. Although most people received stimulus payment during the pandemic, data show that people in higher income groups were less likely to report using their checks to cover food expenses than people in lower income groups [4]. Long-standing and emergency-related social policies are still needed to reduce the mental health disorders caused by lack of food for all populations.

Second, we do not find a statistically significant effect of involuntary unemployment on mental health. By restricting our sample to unemployment from exogenous pandemic-related business shocks, we follow the labor economics literature that uses involuntary job separations to isolate unemployment’s causal impact on health [54,74]. On the one hand, unemployment may increase stressful life events [19] and unstructured time [29]. On the other hand, many generous social policies, such as expanded unemployment insurance and cash transfers, make unemployed people feel less stressful [21,39]. However, other literature points out that mental health disorders are more intense and long-lasting, unemployment insurance benefit might not be adequate to completely mitigate the mental health issues caused by unemployment [31]. Hence, there is still a need for continuing federal and state-level policies to cope with the unemployment and mental health stemming from the spread of disease and economic downturn.

Last, our subgroup results suggest disparate experiences of mental health disorders caused by food insufficiency and economic shocks. Comparison between metro and non-metro areas shows that people living in non-metro areas were more likely to experience mental health disorders due to hunger and income loss. The government and community may provide incentives or subsidized insurance to support less densely populated areas as well as minority communities with limited mental health care access during the crisis to alleviate the effect of hunger and economic shocks [75,76]. For example, literature shows that “Amigas Latinas Motivando el Alma”, a community-based intervention, has been successful in reducing depression among Latina immigrant women [77]. Moreover, digital tools like virtual platforms and apps tailored for specific groups can help people from areas with low access to healthcare service better address mental health disorders [78]. Then, increasing attention could be paid to males’ mental health. Based on our analysis, males experienced greater anxiety than females as a result of food insufficiency. Literature documents that men who experienced hunger or suffer negative economic shocks may be reluctant to seek mental health assistance and avoid expressing emotions [30,79,80]. The heterogeneity observed between males and females calls for targeted mental health care and food assistance, particularly for males, to cope with future economic downturns and public health crises. Overall, governments and policymakers should adopt varied strategies to address the negative impacts of the pandemic, fulfill the needs of different vulnerable populations, and establish an effective and efficient health system in managing external challenges from future public health emergencies [78].

Although this paper provides important new insights about food and mental health hardship during COVID-19, there are several limitations. First, because we do not observe all household members’ mental health, employment status or income, we cannot estimate a collective household model which accounts for intra-household allocation. That being said, our model establishes the relationship between economic shocks, food insufficiency and mental health based on the answers from a representative member of households. Additionally, households in the survey are only identified at the state level, not county or zip-code level so we are unable to identify the local food environment. For the empirical analysis, though the exclusion restriction cannot be tested directly (as in all IV research), we provide extensive statistical evidence and theoretical justification supporting our identifying assumptions.

Our analysis prompts some research questions worth further investigation. For example, one could conduct a survey of time use and individual household members’ information to estimate the relationship between mental health, economic shocks and food insufficiency based on a collective household production model. Another area for future research is to incorporate local food shopping environments such as food retailers, community food assistance such as food pantries or soup kitchens using more detailed data. Because our analysis is based on U.S. survey data, the findings may not generalize to other institutional or cultural contexts. Future research could use comparable real-time survey data from other countries to examine whether the patterns we document hold in different settings.

## Supporting information

S1 File Appendix Tables. (DOCX)
