# Supplementary material for: Economic shocks, food insufficiency and mental health: Evidence from the COVID-19 pandemic
Source: PLoS One. 2026 Mar 12;21(3):e0344745. doi: 10.1371/journal.pone.0344745 (PMC12981442; doi:10.1371/journal.pone.0344745)
Supplement: S1 File — (DOCX) [file pone.0344745.s001.docx]

**S1 File. Appendix Tables**

# Table A1: Summary Statistics of Assistance-Related Variables of Subgroups

|  |  |  |  |  |  |  |  |  |  |
| --- | --- | --- | --- | --- | --- | --- | --- | --- | --- |
| Assistance-related Variables | Mortgage | Rent | P-value | Male | Female | P-value | Non-Metro | Metro | P-value |
|  |  |  |  |  |  |  |  |  |  |
| Free Food | 0.057 | 0.085 | <0.001 | 0.051 | 0.074 | <0.001 | 0.068 | 0.058 | <0.001 |
|  | (0.240) | (0.279) |  | (0.233) | (0.263) |  | (0.254) | (0.245) |  |
| Unemployment Insurance | 0.154 | 0.246 | <0.001 | 0.161 | 0.193 | <0.001 | 0.177 | 0.185 | <0.001 |
|  | (0.387) | (0.456) |  | (0.399) | (0.408) |  | (0.396) | (0.418) |  |
| SNAP | 0.03 | 0.107 | <0.001 | 0.035 | 0.068 | <0.001 | 0.057 | 0.05 | <0.001 |
|  | (0.199) | (0.311) |  | (0.212) | (0.255) |  | (0.238) | (0.240) |  |
| Mental Health Care | 0.112  (0.317) | 0.161  (0.363) | <0.001 | 0.088  (0.291) | 0.145  (0.350) | <0.001 | 0.117  (0.321) | 0.132  (0.339) | <0.001 |

*Notes:* All of assistance-related variables are indicators. Mean of summary statistics show the percent of households in that group receive such assistance. Standard deviations are in parentheses. P-values correspond to t tests of equality of means between groups.

# Table A2-1: Robustness Checks

|  | (1) | (2) | (3) | (4) | (5) | (6) |
| --- | --- | --- | --- | --- | --- | --- |
| Dep. Var: Anxiety  (GAD-2) | No Economic Shocks | No Food Insufficiency | No Income  Residential Mobility | Add COVID Cases Vaccine Rates | HH Member= 1 | HH Member>1 |
|  |  |  |  |  |  |  |
| Food Insufficiency | 0.291*** |  | 0.280*** | 0.266*** | 0.289*** | 0.258*** |
|  | (0.009) |  | (0.012) | (0.012) | (0.041) | (0.012) |
| Income Loss |  | 0.143*** | 0.133*** | 0.128*** | 0.070* | 0.120*** |
|  |  | (0.008) | (0.008) | (0.008) | (0.038) | (0.008) |
| Unemployment |  | 0.047** | -0.020 | -0.015 | 0.041 | 0.001 |
|  |  | (0.021) | (0.023) | (0.024) | (0.075) | (0.023) |
| Household size | 0.009*** | -0.001 | -0.003*** | -0.001 |  | -0.002* |
|  | (0.001) | (0.001) | (0.001) | (0.001) |  | (0.001) |
| Number of kids | -0.019*** | -0.008*** | -0.008*** | -0.010*** |  | -0.009*** |
|  | (0.001) | (0.001) | (0.001) | (0.001) |  | (0.001) |
| Income 25,000-49,999 | 0.007* | -0.002 |  | 0.008** | -0.017 | 0.013*** |
|  | (0.004) | (0.004) |  | (0.004) | (0.010) | (0.005) |
| Income 50,000-99,999 | -0.019*** | -0.036*** |  | -0.011*** | -0.050*** | -0.004 |
|  | (0.004) | (0.004) |  | (0.004) | (0.015) | (0.004) |
| Income 100,000-199,999 | -0.064*** | -0.074*** |  | -0.045*** | -0.071*** | -0.042*** |
|  | (0.005) | (0.004) |  | (0.004) | (0.018) | (0.005) |
| Income >200,000 | -0.098*** | -0.094*** |  | -0.069*** | -0.090*** | -0.067*** |
|  | (0.006) | (0.008) |  | (0.007) | (0.018) | (0.007) |
| Age | 0.001* | 0.002*** | -0.001 | 0.0004 | -0.0001 | 0.001 |
|  | (0.0005) | (0.0004) | (0.0004) | (0.0004) | (0.002) | (0.001) |
| Age square | -0.0005*** | -0.0007*** | -0.0005*** | -0.0005*** | -0.0005** | -0.0005*** |
|  | (0.00001) | (0.00001) | (0.00001) | (0.00001) | (0.00002) | (0.00001) |
| Male | -0.083*** | -0.084*** | -0.087*** | -0.082*** | -0.101*** | -0.079*** |
|  | (0.002) | (0.002) | (0.002) | (0.002) | (0.005) | (0.002) |
| Married | -0.034*** | -0.043*** | -0.046*** | -0.035*** | -0.003 | -0.041*** |
|  | (0.002) | (0.002) | (0.002) | (0.002) | (0.010) | (0.002) |
| Hispanic | -0.035*** | -0.035*** | -0.032*** | -0.038*** | -0.014 | -0.040*** |
|  | (0.005) | (0.004) | (0.004) | (0.004) | (0.009) | (0.004) |
| Black | -0.059*** | -0.042*** | -0.052*** | -0.058*** | -0.063*** | -0.058*** |
|  | (0.004) | (0.004) | (0.004) | (0.004) | (0.010) | (0.004) |
| Asian | -0.082*** | -0.078*** | -0.075*** | -0.077*** | -0.075*** | -0.078*** |
|  | (0.005) | (0.005) | (0.005) | (0.005) | (0.016) | (0.004) |
| Other Race | 0.016*** | 0.022*** | 0.015*** | 0.012*** | 0.0004 | 0.015** |
|  | (0.004) | (0.004) | (0.004) | (0.004) | (0.018) | (0.006) |
| HS degree or GED | -0.007 | -0.018 | -0.005 | -0.003 | -0.013 | -0.003 |
|  | (0.010) | (0.012) | (0.010) | (0.011) | (0.049) | (0.013) |
| Some college/AA degree | 0.027** | 0.005 | 0.023** | 0.030*** | 0.014 | 0.032** |
|  | (0.011) | (0.013) | (0.011) | (0.012) | (0.050) | (0.014) |
| Bachelors’/Graduate degree | 0.014 | -0.006 | 0.008 | 0.028** | 0.014 | 0.029** |
|  | (0.011) | (0.012) | (0.011) | (0.011) | (0.049) | (0.013) |
| Residential Mobility | -0.0003 | -0.001 |  | -0.0007 | 0.001 | -0.001 |
|  | (0.001) | (0.001) |  | (0.001) | (0.001) | (0.001) |
| Stay-at-home order | -0.006 | -0.005 | -0.005 | -0.007** | -0.003 | -0.006 |
|  | (0.009) | (0.010) | (0.009) | (0.003) | (0.010) | (0.010) |
| Non-essential-business | -0.006 | -0.006 | 0.001 | 0.001 | 0.001 | -0.006 |
|  | (0.007) | (0.007) | (0.006) | (0.006) | (0.011) | (0.008) |
| Time trend | 0.001*** | 0.002*** | 0.001*** | 0.002*** | -0.003*** | 0.002*** |
|  | (0.00007) | (0.0002) | (0.0003) | (0.0004) | (0.0004) | (0.0002) |
| COVID Cases per capita |  |  |  | 40.902*** |  |  |
|  |  |  |  | (6.991) |  |  |
| Vaccine rate |  |  |  | -0.001*** |  |  |
|  |  |  |  | (0.00001) |  |  |
| Constant | 0.420*** | 0.405*** | 0.414*** | 0.370*** | 0.518*** | 0.369*** |
|  | (0.015) | (0.015) | (0.015) | (0.015) | (0.067) | (0.015) |
|  |  |  |  |  |  |  |
| Observations | 1,039,923 | 1,039,923 | 1,039,923 | 1,039,923 | 141,201 | 898,722 |

*Notes*: All estimation results are based on linear probability models with instrumental variables and adjusted by sampling weights. Standard errors are clustered at state level. ***, **, and *, denote significance at the 1%, 5%, and 10% levels, respectively.

# Table A2-2: Robustness Checks

|  | (1) | (2) | (3) | (4) | (5) | (6) |
| --- | --- | --- | --- | --- | --- | --- |
| Dep. Var:  Depression  (PHQ-2) | No Economic Shocks | No Food Insufficiency | No Income  Residential Mobility | Add COVID Cases Vaccine Rates | HH Member= 1 | HH Member>1 |
|  |  |  |  |  |  |  |
| Food Insufficiency | 0.257*** |  | 0.253*** | 0.235*** | 0.282*** | 0.229*** |
|  | (0.015) |  | (0.012) | (0.012) | (0.041) | (0.012) |
| Income Loss |  | 0.122*** | 0.116*** | 0.109*** | 0.067 | 0.109*** |
|  |  | (0.010) | (0.009) | (0.009) | (0.055) | (0.008) |
| Unemployment |  | 0.047** | -0.010 | -0.007 | 0.038 | 0.009 |
|  |  | (0.018) | (0.017) | (0.018) | (0.081) | (0.018) |
| Household size | 0.007*** | -0.001 | -0.003** | -0.001 |  | -0.0002 |
|  | (0.001) | (0.002) | (0.001) | (0.001) |  | (0.002) |
| Number of kids | -0.023*** | -0.013*** | -0.013*** | -0.015*** |  | -0.015*** |
|  | (0.001) | (0.001) | (0.001) | (0.001) |  | (0.001) |
| Income 25,000-49,999 | 0.007* | -0.0002 |  | 0.009** | -0.005 | 0.013** |
|  | (0.004) | (0.005) |  | (0.004) | (0.012) | (0.005) |
| Income 50,000-99,999 | -0.031*** | -0.046*** |  | -0.023*** | -0.039** | -0.019*** |
|  | (0.003) | (0.004) |  | (0.004) | (0.018) | (0.004) |
| Income 100,000-199,999 | -0.071*** | -0.080*** |  | -0.055*** | -0.063*** | -0.051*** |
|  | (0.004) | (0.004) |  | (0.004) | (0.022) | (0.004) |
| Income >200,000 | -0.101*** | -0.098*** |  | -0.076*** | -0.075*** | -0.072*** |
|  | (0.005) | (0.006) |  | (0.006) | (0.023) | (0.006) |
| Age | -0.004*** | -0.002*** | -0.005*** | -0.004*** | -0.002 | -0.005*** |
|  | (0.001) | (0.001) | (0.001) | (0.001) | (0.002) | (0.001) |
| Age square | 0.0000001 | -0.00001** | 0.00002*** | 0.00001 | -0.00002 | 0.00002** |
|  | (0.00001) | (0.00001) | (0.00001) | (0.00001) | (0.00002) | (0.00001) |
| Male | -0.030*** | -0.031*** | -0.034*** | -0.029*** | -0.046*** | -0.028*** |
|  | (0.002) | (0.002) | (0.002) | (0.002) | (0.005) | (0.002) |
| Married | -0.053*** | -0.060*** | -0.066*** | -0.053*** | -0.008 | -0.055*** |
|  | (0.003) | (0.003) | (0.002) | (0.002) | (0.011) | (0.003) |
| Hispanic | -0.031*** | -0.031*** | -0.026*** | -0.033*** | -0.022*** | -0.035*** |
|  | (0.004) | (0.004) | (0.003) | (0.004) | (0.008) | (0.004) |
| Black | -0.035*** | -0.020*** | -0.028*** | -0.035*** | -0.055*** | -0.032*** |
|  | (0.006) | (0.005) | (0.006) | (0.006) | (0.009) | (0.006) |
| Asian | -0.032*** | -0.029*** | -0.025*** | -0.028*** | -0.034*** | -0.028*** |
|  | (0.005) | (0.004) | (0.005) | (0.005) | (0.010) | (0.006) |
| Other Race | 0.019*** | 0.024*** | 0.019*** | 0.016*** | 0.006 | 0.017*** |
|  | (0.004) | (0.004) | (0.004) | (0.004) | (0.013) | (0.004) |
| HS degree or GED | -0.005 | -0.014 | -0.004 | -0.0005 | -0.024 | 0.003 |
|  | (0.009) | (0.010) | (0.009) | (0.009) | (0.045) | (0.010) |
| Some college/AA degree | 0.017* | -0.002 | 0.010 | 0.020** | 0.009 | 0.022* |
|  | (0.009) | (0.011) | (0.009) | (0.010) | (0.043) | (0.012) |
| Bachelors’/Graduate degree | -0.026*** | -0.044*** | -0.037*** | -0.015 | -0.030 | -0.011 |
|  | (0.009) | (0.011) | (0.010) | (0.010) | (0.044) | (0.011) |
| Residential Mobility | 0.002*** | 0.002*** |  | 0.001*** | 0.003*** | 0.002*** |
|  | (0.0005) | (0.001) |  | (0.0005) | (0.001) | (0.0005) |
| Stay-at-home order | -0.002 | -0.0004 | -0.001 | -0.001 | 0.006 | -0.002 |
|  | (0.006) | (0.006) | (0.006) | (0.004) | (0.014) | (0.006) |
| Non-essential-business | -0.003 | -0.003 | -0.002 | 0.00004 | 0.003 | -0.003 |
|  | (0.005) | (0.004) | (0.004) | (0.004) | (0.013) | (0.005) |
| Time trend | 0.002*** | 0.003*** | 0.002*** | 0.002*** | -0.005*** | 0.004*** |
|  | (0.00006) | (0.0001) | (0.0001) | (0.0002) | (0.001) | (0.0001) |
| COVID Cases per capita |  |  |  | 37.184*** |  |  |
|  |  |  |  | (7.391) |  |  |
| Vaccine rate |  |  |  | -0.0004*** |  |  |
|  |  |  |  | (0.0002) |  |  |
| Constant | 0.418*** | 0.404*** | 0.418*** | 0.388*** | 0.515*** | 0.364*** |
|  | (0.016) | (0.016) | (0.016) | (0.017) | (0.073) | (0.017) |
|  |  |  |  |  |  |  |
| Observations | 1,039,923 | 1,039,923 | 1,039,923 | 1,039,923 | 141,201 | 898,722 |

*Notes*: All estimation results are based on linear probability models with instrumental variables and adjusted by sampling weights. Standard errors are clustered at state level. ***, **, and *, denote significance at the 1%, 5%, and 10% levels, respectively.

# Table A3: Estimates of LPM-IV models by Food Insufficiency Conditions

|  | (1) | (2) | (3) | (4) |
| --- | --- | --- | --- | --- |
| Dep. Variable: | Anxiety  (GAD-2) | Anxiety  (GAD-2) | Depression (PHQ-2) | Depression (PHQ-2) |
|  |  |  |  |  |
| Current FI, No prior FI | 0.291*** |  | 0.238*** |  |
|  | (0.018) |  | (0.021) |  |
| Current and Prior FI |  | 0.491*** |  | 0.427*** |
|  |  | (0.029) |  | (0.022) |
| Income Loss | 0.135*** | 0.129*** | 0.112*** | 0.110*** |
|  | (0.007) | (0.008) | (0.009) | (0.009) |
| Unemployment | 0.051*** | 0.064*** | -0.017 | -0.008 |
|  | (0.004) | (0.004) | (0.024) | (0.018) |
| Constant | 0.393*** | 0.355*** | 0.392*** | 0.362*** |
|  | (0.015) | (0.016) | (0.016) | (0.017) |
|  |  |  |  |  |
| Observations | 1,001,449 | 1,039,923 | 1,001,449 | 1,039,923 |

Notes: The “Current FI, No prior FI” category indicates households that were food-insufficient at the time of the survey (past seven days) but not prior to March 13, 2020. “Current and Prior FI” indicates households that were food-insufficient both at the time of the survey and before March 13, 2020 (n = 24,314, representing 39% of all food-insufficient households). The reference group for both FI categories is households with no food insufficiency. All estimation results are based on linear probability models with instrumental variables and adjusted by sampling weights. Standard errors are clustered at state level. ***, **, and *, denote significance at the 1%, 5%, and 10% levels, respectively. All models include the same control variables as Table 2.

# Table A4: Programs Comparison

|  | (1)  Anxiety | (2)  Anxiety | (3)  Depression | (4)  Depression |
| --- | --- | --- | --- | --- |
| Dep. Var: | (GAD-2) | (GAD-2) | (PHQ-2) | (PHQ-2) |
|  |  |  |  |  |
| Food Insufficiency | 0.272*** | 0.266*** | 0.274*** | 0.262*** |
|  | (0.008) | (0.006) | (0.010) | (0.005) |
| Income loss | 0.127*** | 0.128*** | 0.093*** | 0.109*** |
|  | (0.008) | (0.008) | (0.011) | (0.009) |
| Current unemployed | 0.044 | -0.019 | 0.004 | -0.012 |
|  | (0.040) | (0.023) | (0.047) | (0.019) |
| FI*Receive SNAP | -0.052*** |  | -0.041*** |  |
|  | (0.010) |  | (0.012) |  |
| FI*Receive Free Food |  | -0.045*** |  | -0.045*** |
|  |  | (0.012) |  | (0.012) |
| Household size | -0.004*** | -0.001 | -0.0002 | -0.001 |
|  | (0.001) | (0.001) | (0.002) | (0.001) |
| Number of kids | -0.007*** | -0.011*** | -0.016*** | -0.016*** |
|  | (0.002) | (0.001) | (0.002) | (0.001) |
| Income 25,000-49,999 | 0.019*** | 0.007* | 0.017*** | 0.009** |
|  | (0.005) | (0.004) | (0.006) | (0.004) |
| Income 50,000-99,999 | 0.012*** | -0.011*** | -0.004 | -0.020*** |
|  | (0.005) | (0.004) | (0.004) | (0.003) |
| Income 100,000-199,999 | -0.021*** | -0.044*** | -0.034*** | -0.051*** |
|  | (0.004) | (0.004) | (0.004) | (0.003) |
| Income >200,000 | -0.045*** | -0.068*** | -0.058*** | -0.071*** |
|  | (0.006) | (0.007) | (0.005) | (0.005) |
| Age | -0.002*** | 0.0003 | -0.007*** | -0.004*** |
|  | (0.001) | (0.0004) | (0.001) | (0.001) |
| Age square | -0.00002*** | -0.00005*** | 0.00004*** | 0.00001* |
|  | (0.00001) | (0.00001) | (0.00001) | (0.00001) |
| Male | -0.088*** | -0.082*** | -0.032*** | -0.029*** |
|  | (0.002) | (0.002) | (0.001) | (0.002) |
| Married | -0.036*** | -0.035*** | -0.054*** | -0.053*** |
|  | (0.002) | (0.002) | (0.003) | (0.002) |
| Hispanic | -0.036*** | -0.039*** | -0.031*** | -0.035*** |
|  | (0.004) | (0.004) | (0.004) | (0.004) |
| Black | -0.063*** | -0.058*** | -0.032*** | -0.036*** |
|  | (0.004) | (0.004) | (0.005) | (0.005) |
| Asian | -0.089*** | -0.077*** | -0.029*** | -0.028*** |
|  | (0.003) | (0.005) | (0.005) | (0.005) |
| Other Race | 0.006 | 0.013*** | 0.013*** | 0.015*** |
|  | (0.005) | (0.004) | (0.005) | (0.004) |
| HS degree or GED | -0.002 | -0.002 | 0.007 | 0.003 |
|  | (0.012) | (0.010) | (0.015) | (0.009) |
| Some college/AA degree | 0.042*** | 0.031*** | 0.031** | 0.024** |
|  | (0.013) | (0.011) | (0.015) | (0.010) |
| Bachelors’/Graduate degree | 0.047*** | 0.028*** | 0.001 | -0.009 |
|  | (0.012) | (0.010) | (0.015) | (0.010) |
| Residential Mobility | 0.004*** | -0.0004 | 0.004*** | 0.002*** |
|  | (0.001) | (0.0005) | (0.001) | (0.0005) |
| Stay-at-home order | 0.022* | -0.005 | 0.023*** | -0.0002 |
|  | (0.012) | (0.009) | (0.003) | (0.006) |
| Non-essential-business | -0.022** | -0.005 | -0.025*** | -0.002 |
|  | (0.010) | (0.007) | (0.008) | (0.004) |
| Time trend | -0.002*** | 0.001*** | 0.001*** | 0.002*** |
|  | (0.0004) | (0.0002) | (0.0003) | (0.0001) |
| Constant | 0.462*** | 0.387*** | 0.453*** | 0.383*** |
|  | (0.018) | (0.014) | (0.018) | (0.016) |
|  |  |  |  |  |
| Observations | 540,064 | 1,038,654 | 540,064 | 1,038,654 |

*Notes*: All estimation results are based on linear probability models with instrumental variables and adjusted by sampling weights. Standard errors are clustered at state level. ***, **, and *, denote significance at the 1%, 5%, and 10% levels, respectively.

# Table B Descriptions of variables

| Variable Name | Brief Definition | Min  Value | Max  Value |
| --- | --- | --- | --- |
| **Dependent Variable** |  |  |  |
| Anxiety (GAD-2) | Whether the respondent felt anxious more than half or nearly every day in the last 7 days | 0 | 1 |
| Depression (PHQ-2) | Whether the respondent felt depressed more than half or nearly every day in the last 7 days | 0 | 1 |
| **FI and Economic Shocks** |  |  |  |
| Food insufficiency | Whether adults in households sometimes or often did not have enough to eat in the last 7 days | 0 | 1 |
| Income loss | Whether the respondent or someone in their household experienced a loss of employment income since March 13, 2020. | 0 | 1 |
| Unemployment | Whether the respondent was employed in the last 7 days | 0 | 1 |
| **Household Characteristics** |  |  |  |
| Household size | How many members are there in the respondent's household? | 1 | 10 |
| Number of children | How many children are there in the respondent's household? | 0 | 5 |
| Income <$25,000 | Whether the household income was less than $25,000 in 2019 | 0 | 1 |
| Income $25,000-$49,999 | Whether the household income was equal to or greater than $25,000 but less than $50,000 in 2019 | 0 | 1 |
| Income $50,000 - $99,999 | Whether the household income was equal to or greater than $50,000 but less than $100,000 in 2019 | 0 | 1 |
| Income $100,000-$199,999 | Whether the household income was equal to or greater than $100,000 but less than $200,000 in 2019 | 0 | 1 |
| Income >$200,000 | Whether the household income was greater than $200,000 in 2019 | 0 | 1 |
| **Respondent Characteristics** |  |  |  |
| Age | The age of the respondent | 18 | 65 |
| Age square | The square of the respondent's age | 324 | 4225 |
| Male | It is 1 if the respondent is male, 0 if the respondent is female | 0 | 1 |
| Married | It is 1 if the respondent is married, 0 if the respondent is not married (widowed, divorced, separated, never married) | 0 | 1 |
| Hispanic | Whether the respondent is Hispanic | 0 | 1 |
| White | Whether the respondent is non-Hispanic white | 0 | 1 |
| Black | Whether the respondent is non-Hispanic black | 0 | 1 |
| Asian | Whether the respondent is non-Hispanic Asian | 0 | 1 |
| Other | Whether the respondent belongs to a race group other than Hispanic, white, black, or Asian | 0 | 1 |
| Less than high school | Whether the respondent’s highest education level is less than high school | 0 | 1 |
| High school graduate or equivalent | Whether the respondent’s highest education level is high school graduate or equivalent | 0 | 1 |
| Some college or Associates degree | Whether the respondent’s highest education level is some college or an associate’s degree | 0 | 1 |
| Bachelor's or graduate degree | Whether the respondent’s highest education level is a bachelor's or graduate degree | 0 | 1 |
| **State Characteristics** |  |  |  |
| Residential mobility | Mobility trends for places of residence in the respondent's residential state compared to Jan 3–Feb 6, 2020 | 2 | 29 |
| Stay-at-home order | Whether the respondent's residential state implemented the stay-at-home order during the survey week | 0 | 1 |
| Non-essential-business order | Whether the respondent's residential state closed non-essential businesses during the survey week | 0 | 1 |
| Observations | 1,039,923 |  |  |
